# Beyond the digital panopticon: a mixed-methods examination of teacher burnout, psychological capital, and well-being through the lens of work-life balance

**DOI:** 10.3389/fpsyg.2026.1878063

**Published:** 2026-08-26

**Authors:** Chen Cheng, Anurak Panyanuwat, Xiaohong Li, Jie Yang

**Affiliations:** 1 Interdisciplinary Studies College, Payap University, Chiang Mai, Thailand; 2 Human Resources Management Department, Sanda University, Shanghai, China; 3 School of Education Science, Guangxi MinZu University, Nanning, China; 4 College of Education, Shanghai Jian Qiao University, Shanghai, China

**Keywords:** digital involution, mixed-methods, psychological capital (PsyCap), psychological well-being, teacher burnout, work-life balance

## Abstract

**Introduction:**

In the contemporary Chinese educational landscape, characterized by involution (*neijuan*) and increasing digital encroachment, the maintenance of educator well-being has become a critical challenge. This study investigated how teacher burnout and psychological capital (PsyCap) predict psychological well-being (PWB), with a specific focus on the mediating role of work-life balance (WLB).

**Methods:**

An explanatory sequential mixed-methods design was employed. In the quantitative phase, survey data were collected from 842 primary and secondary school teachers across three Chinese provinces and analyzed using Structural Equation Modeling (SEM). In the qualitative phase, the Critical Incident Technique (CIT) was used with 20 teachers selected through deviant case sampling.

**Results:**

SEM revealed that WLB partially mediated the relationships between both burnout (*β* = −0.090) and PsyCap (*β* = 0.122) and PWB. The model accounted for 52.8% of the variance in teacher well-being. Analysis of 40 critical incidents revealed that successful WLB was driven by “tactical pathway generation” rather than passive optimism. However, qualitative narratives also exposed a “digital panopticon” fueled by WeChat-based expectations of 24/7 availability, which led to significant boundary thinning. Furthermore, a “competence trap” was identified, where high self-efficacy paradoxically accelerated burnout by encouraging teachers to accept excessive invisible labor.

**Discussion:**

These findings suggest that while PsyCap is a vital personal resource, its effectiveness is constrained by structural and digital boundary fractures within the Chinese school system. Implications for school leadership and policy interventions aimed at protecting teacher mental health in the digital age are discussed.

## 1 Introduction

China’s educational landscape is characterized by involution (neijuan), a state where escalating effort yields diminishing institutional returns. This atmosphere of irrational competition prioritizes performance metrics at the expense of educator health (Cheng and Diao, 2024). Traditional Confucian ideals of selfless dedication are being supplanted by “involution anxiety” and internalized failure (Wang and Yang, 2024; Xia et al., 2025), transforming the maintenance of psychological well-being (PWB) from a professional goal into a fundamental survival challenge.

Digital technology has accelerated this decline by eroding work-life boundaries. Super-apps such as WeChat have institutionalized a “digital panopticon” that demands 24/7 availability (Tian, 2020; Yu, 2024). Such constant connectivity results in “recovery obstruction,” hindering the psychological detachment necessary for emotional replenishment (Derks et al., 2014; Sonnentag and Fritz, 2015). Ultimately, this digital shift generates a chronic “time poverty” that fuels exhaustion and cynicism (Zhu et al., 2025).

While the Job Demands-Resources (JD-R) model identifies job demands as the primary driver of health impairment (Bakker and Demerouti, 2017), the function of psychological capital (PsyCap)—comprising self-efficacy, hope, resilience, and optimism—remains complex within the Chinese context (Luthans et al., 2015; Xanthopoulou et al., 2007). Crucially, high efficacy may paradoxically trigger a “competence trap,” wherein capable teachers are burdened with excessive “invisible labor” that accelerates burnout (Karasek and Theorell, 1990; Ye et al., 2026). This implies that personal resources are not stand-alone assets but are filtered through an individual’s work-life balance (WLB). Work-life balance represents the primary friction point between structural pressures and individual agency. Although WLB involves role engagement and perceived control (Casper et al., 2018), cultural mandates of self-sacrifice often frame boundary-setting as a professional failure, leading to significant “boundary thinning” (Wu et al., 2025). Investigating these dynamics requires an approach that captures broad statistical trends alongside the behavioral nuances of real-time conflict.

Despite extensive research on teacher stress, mixed-methods evidence is scarce regarding how PsyCap and burnout interact within digital surveillance frameworks. Existing literature relies heavily on cross-sectional surveys that overlook the tactical strategies employed during specific work-life “friction points” (Luthans and Youssef-Morgan, 2017; Newman et al., 2014). Furthermore, the “paradox of efficacy” in the involution context remains under-examined. The present study addresses these gaps through an explanatory sequential mixed-methods design. First, Structural Equation Modeling (SEM) is used to test a mediation model among 842 teachers. Subsequently, the Critical Incident Technique (CIT) is applied to interview 20 “deviant cases” whose outcomes contradicted their psychological profiles. By analyzing concrete incidents of boundary enactment, this research provides a nuanced understanding of well-being “beyond the digital panopticon” in modern China.

## 2 Literature review

### 2.1 Teacher burnout and psychological well-being in the era of involution

Contemporary Chinese education is increasingly defined by involution, a socio-cultural phenomenon characterized by intensive, irrational competition (Cheng and Diao, 2024). Conceptually, this dynamic forces educators to exert escalating effort for diminishing returns on personal and institutional investment. This cycle often precipitates “involution anxiety”—a state of defensive competition and chronic exhaustion that hinders the internalization of professional goals (Wang and Yang, 2024; Ye et al., 2026). Within this landscape, a confluence of systemic performance metrics and a cultural ethos of selfless dedication dictates how teachers expend and recover their personal resources (Liu and Onwuegbuzie, 2012; Xia et al., 2025).

Burnout serves as the proximal mechanism linking these structural pressures to adverse outcomes. As a psychological syndrome, it comprises emotional exhaustion, depersonalization (cynicism), and reduced personal accomplishment (Maslach and Jackson, 1981; Maslach et al., 1996, 2001; Schaufeli and Bakker, 2004). In involuted settings, title-oriented evaluation systems catalyze these symptoms by compelling teachers to pursue validated status markers through ever-increasing outputs (Cheng and Diao, 2024). Consequently, educators may transition from professional involvement to defensive coping, eventually culminating in clinical burnout or strategic “lying flat” (Cheng and Diao, 2024). Such states lead to diminished sympathy for students, weakened involvement, and lower productivity (Vandenberghe, 1999), with early exhaustion serving as a robust predictor of subsequent psychopathological symptoms (Burić et al., 2019).

Burnout undermines psychological well-being by depleting the capacities essential for eudaimonic functioning (Ryff, 1989, 1995, 2014; Skaalvik and Skaalvik, 2017). Emotional exhaustion impairs environmental mastery and flexibility, while depersonalization erodes positive relations needed for a supportive classroom (Klusmann et al., 2016; Ryff, 2014). Reduced personal accomplishment further weakens self-acceptance and purpose, especially when teacher identity is tied to institutional evaluations (Skaalvik and Skaalvik, 2010, 2014). Empirical evidence confirms that these burnout dimensions significantly impair all six facets of well-being among Chinese educators (Ma and Wu, 2024). Although structural involution affects the workforce broadly, with sex differences often negligible (Kaur and Singh, 2014), male teachers and class tutors face heightened vulnerability (Wei and Ye, 2022). Conversely, a strong career calling can buffer against these pressures and sustain well-being (Zhao et al., 2022).

The heightened vulnerability of class tutors (banzhuren) stems from their unique role as the primary liaison among the school, students, and parents. Unlike subject teachers, they bear continuous responsibility for welfare, behavior, and academic management that extends well beyond the school day. The Job Demands-Resources (JD-R) model clarifies this exhaustion: tutors face intensified emotional demands from managing parental expectations and student conflicts (Bakker and Demerouti, 2017). This is compounded by digital platforms like WeChat, which have forced tutors into permanent availability. Parents routinely contact them after hours, creating chronic boundary permeability that prevents psychological detachment (Derks et al., 2014; Sonnentag and Fritz, 2015; Tian, 2020; Wu et al., 2025). Coupled with direct accountability for student performance metrics, this boundary-blurring role leads to higher burnout rates than subject specialists (Wei and Ye, 2022).

Male teachers, though less studied, face distinct sociocultural pressures. As a numerical minority in a predominantly female workforce, they encounter expectations tied to traditional Confucian gender norms, where teaching is often framed as a lower-prestige “feminine” profession, creating role strain (Liu and Onwuegbuzie, 2012). They also face intensified scrutiny in child-facing roles, which fuels hypervigilance, and are more often assigned to administrative or disciplinary tasks. Limited same-gender peer networks further reduce mentorship and social buffering (Hobfoll et al., 2018). From a Conservation of Resources (COR) perspective, these combined pressures amount to a gender-specific resource drain, making male teachers particularly susceptible to burnout without sufficient organizational support (Hobfoll, 2002).

The Job Demands-Resources (JD-R) model clarifies how these conditions initiate a health-impairment process (Bakker and Demerouti, 2014, 2017; Demerouti et al., 2001; Schaufeli and Taris, 2014). High demands—including heavy workloads, time pressure, and the emotional labor of stakeholder management—deplete energy reserves and lead to exhaustion (Hakanen et al., 2006; Kyriacou, 2001). Unsupportive workplace milieus, marginalization, or bullying further exacerbate this decline (Sohail et al., 2023). In the current digital shift, technology-enhanced instruction has introduced “time poverty,” where temporal scarcity fully mediates well-being declines through exhaustion (Zhu et al., 2025).

These stressors often precipitate work–family conflict, damaging perceived work ability (Zábrodská et al., 2024). While the JD-R health-impairment process is universal, it is locally moderated by digital encroachment and cultural context (Bakker et al., 2023; Wang et al., 2025). In China, high-stakes exam structures, Confucian duties, and 24/7 connectivity through super-apps erode traditional professional boundaries (Wang et al., 2025). Because organizational climate and resource availability determine whether this decline is accelerated or buffered (Angelini et al., 2024; Md Shah et al., 2024), identifying intervention points like work-life balance is essential in an increasingly involuted system.

### 2.2 Psychological capital (PsyCap): dimensions, mechanisms, and educator resilience

As a higher-order, state-like construct, psychological capital (PsyCap) fosters psychological thriving and mitigates the resource depletion associated with burnout (Luthans, 2002a, 2002b; Luthans et al., 2015). Distinct from human or social capital, PsyCap focuses on “who you are”—developable internal capacities that drive positive growth (Luthans et al., 2004; Luthans and Youssef, 2004; Seligman and Csikszentmihalyi, 2000). The construct is traditionally operationalized through four synergistic dimensions—self-efficacy, hope, resilience, and optimism (HERO)—which sustain work engagement and well-being (Luthans et al., 2007a, 2007b; Luthans and Youssef-Morgan, 2017; Newman et al., 2014). Recent conceptual expansions have further integrated grit for long-term persistence and identified synergistic benefits when PsyCap is coupled with effective emotion regulation strategies (Qiu et al., 2025; Shao, 2023).

Within the Job Demands-Resources (JD-R) framework, these personal resources fuel motivational pathways that bolster perceived control and arrest the health-impairment process (Bakker and Demerouti, 2014, 2017; Schaufeli and Taris, 2014; Xanthopoulou et al., 2007, 2009). Consistent evidence across the Chinese healthcare and education sectors links higher PsyCap to reduced burnout and greater institutional commitment (Avey et al., 2009, 2011; Hazan-Liran and Karni-Vizer, 2024; Luthans et al., 2005). Network analyses further identify hope and optimism as critical “bridge symptoms” that can halt burnout progression within the teacher well-being network (Xue et al., 2023). According to conservation of resources (COR) theory, educators with robust PsyCap are better positioned to mobilize support, thereby avoiding the “loss spirals” inherent in chronic exhaustion (Hobfoll, 2001, 2002; Hobfoll et al., 2018).

The efficacy of PsyCap is frequently shaped by cognitive and organizational variables. High resource levels foster mindfulness and adaptive coping, which diminish burnout—especially among veteran educators (Liu and Du, 2024). While PsyCap typically buffers work–family conflict (Pu et al., 2017), variables such as lack of structural empowerment or heightened political tension can stifle its protective capacity (Kaya and Altınkurt, 2018; Zhang and Li, 2025). Even in high-burnout specialized settings, personal assets remain insufficient without corresponding institutional support (Hazan-Liran and Karni-Vizer, 2024; Wang et al., 2025).

Despite its theoretical strength, current research is limited by a reliance on cross-sectional surveys that capture static beliefs rather than behavioral enactment (Luthans and Youssef-Morgan, 2017; Newman et al., 2014). Translating internal resources into action requires proactive negotiation or psychological detachment (Bandura, 1997, 2001; Klusmann et al., 2016; Skaalvik and Skaalvik, 2014, 2017). By employing the Critical Incident Technique (CIT), this study addresses this gap by examining how PsyCap dimensions are actually operationalized during specific work-life friction points, rather than relying solely on generalized self-reports of resource availability.

### 2.3 Work-life balance (WLB) as a mediating mechanism

The connection between psychological predictors and educator well-being is predominantly indirect, filtered through the capacity to harmonize competing life roles (Bakker and Demerouti, 2017; Derakhshan and Fathi, 2024; Hobfoll, 2002). This capacity, defined as work-life balance (WLB), has transitioned from the mere absence of role conflict into a multifaceted construct involving role engagement, satisfaction, and perceived control (Casper et al., 2018; Greenhaus and Allen, 2011; Kalliath and Brough, 2008). Modern scholarship emphasizes a reciprocal harmony where professional and personal activities coexist without chronic mutual detriment (Fisher et al., 2009; Haar et al., 2014), leading to the search for unifying frameworks like the PERMA-H model to address escalating teacher stress and turnover (Avola et al., 2025; Bisht et al., 2025).

Within the teaching profession, WLB is best conceptualized along a conflict-enrichment continuum (Hayman, 2005). While traditional markers like Work Interference with Personal Life (WIPL) and Personal Life Interference with Work (PLIW) assess role strain, Work-Personal Life Enhancement (WPLE) captures how professional achievements bolster personal vitality (Agha, 2017; Carlson et al., 2000; Cinamon and Rich, 2005; Fisher et al., 2009). This interface serves as a comprehensive indicator of recovery potential and affective spillover, directly influencing well-being and professional identity (Haar et al., 2014; Sirgy and Lee, 2018; Sun et al., 2022). Furthermore, these outcomes are modulated by a teacher’s specific reactive or proactive coping strategies in the face of job demands (Bermejo-Toro et al., 2016).

Mediation occurs because psychological resources and occupational strains influence the functional boundaries of a person’s life before impacting overall well-being (Demerouti et al., 2015; Haar et al., 2014; Hakanen et al., 2006; Sonnentag and Fritz, 2015; Xanthopoulou et al., 2009). High psychological capital (PsyCap) empowers teachers to negotiate these boundaries through self-efficacy, tactical hope, resilience, and optimism (Bandura, 1997; Luthans et al., 2006, 2015; Masten, 2014; Seligman, 1998; Snyder, 2002). This proactive management facilitates “work thriving,” which enhances classroom effectiveness while reducing turnover intentions (Cho et al., 2025; Johari et al., 2018; Zhang and Li, 2025; Zhao et al., 2024).

Conversely, burnout inhibits balance by depleting the energetic reserves required for boundary maintenance (Maslach et al., 2001; Schaufeli and Bakker, 2004). Emotional exhaustion triggers work-related rumination, while depersonalization diminishes the motivation for role negotiation (Bakker and Demerouti, 2017; Maslach and Leiter, 2016; Sonnentag and Fritz, 2015). In high-stress pedagogical settings, poor WLB serves as a proximal cause for clinical distress and lower life satisfaction (Xanthopoulou et al., 2009).

Boundary and border theories explain this mediation by positing that well-being depends on the flexibility and permeability of the lines separating life domains (Ashforth et al., 2000; Bakker and Demerouti, 2014, 2017; Clark, 2000; Kreiner, 2006). In the Chinese educational context, individual preferences are frequently overridden by a “digital panopticon” fueled by WeChat-based expectations of constant availability (Desrochers and Morgan, 2021; Kreiner et al., 2009; Nippert-Eng, 1996; Tian, 2020; Yu, 2024). This environment facilitates chronic boundary thinning and resource-draining role transitions that neutralize personal agency (Ashforth et al., 2000; Bandura, 2001; Derks et al., 2014; Wu et al., 2025). Thus, utilizing the Critical Incident Technique is essential to capture how teachers attempt boundary enactment amidst these digital friction points (Ashforth et al., 2000; Kreiner, 2006; Yu, 2024).

### 2.4 Digital encroachment and the “digital panopticon”

Traditional WLB frameworks, including boundary theory and the JD-R model (Ashforth et al., 2000; Bakker and Demerouti, 2017; Clark, 2000), must now account for the systematic erosion of temporal and spatial borders caused by mobile super-apps (Tian, 2020; Yu, 2024). Within the Chinese educational context, after-hours ICT usage is driven by institutional performance expectations and individual integration preferences (Bauwens et al., 2020), positioning teacher well-being within rigid digital and organizational structures (Dávila et al., 2024). This “digital panopticon” enables continuous surveillance by administrators and parents, forcing educators to self-police their responsiveness to avoid the professional penalties of digital silence (Foucault, 1977; Tian, 2020; Yu, 2024).

The mechanisms through which digital encroachment damages well-being are multifaceted. First, the constant connectivity generates anticipatory stress, where teachers remain psychologically “on call” even during designated personal time. Anticipating work-related notifications disrupts the cognitive disengagement necessary for recovery (Derks et al., 2014; Sonnentag and Fritz, 2015). This state of hypervigilance has been shown to impair sleep quality, increase physiological stress responses, and reduce the restorative value of leisure activities (Jeon et al., 2018). Second, digital platforms create performative pressure—teachers feel compelled to demonstrate their dedication through rapid responses to parent or administrator messages, as non-response may be interpreted as negligence or lack of commitment (Jian and Zhou, 2025; Wu et al., 2025).

Third, the blurring of professional and personal communication channels, particularly through WeChat where work contacts often co-exist with personal relationships, reduces the psychological distance between domains (Tian, 2020). The same platform used for family communication also delivers grading updates and parent complaints, making true detachment nearly impossible. This “technological integration” is distinct from voluntary segmentation preferences; it is structurally imposed rather than individually chosen (Bauwens et al., 2020). According to Conservation of Resources (COR) theory, this constant vigilance creates a severe resource drain by demanding energy for both pedagogical tasks and perpetual alertness (Hobfoll, 2002; Hobfoll et al., 2018).

The digital panopticon also generates a distinct form of “time poverty” that lacks the fixed boundaries of classroom teaching; digital demands extend limitlessly into evenings, weekends, and holidays (Zhu et al., 2025). Teachers report a dramatic increase in after-hours communication, yet recovery has proportionally diminished as the work-life boundary dissolves (Fang et al., 2024). Digital permeability predicts burnout and turnover intentions as strongly as—or more than—traditional workload measures, suggesting that digital encroachment represents a qualitatively different job demand that undermines the very possibility of psychological detachment (Wu et al., 2025). This trend is further intensified by technostress and work values that prioritise unconditional engagement (Fang et al., 2024; Jian and Zhou, 2025; Wu et al., 2025).

### 2.5 Boundary theory as a framework for understanding digital permeability

To understand how digital encroachment damages teacher well-being, it is necessary to turn to Boundary Theory, also known as Work-Family Border Theory (Ashforth et al., 2000; Clark, 2000). This framework provides a language for describing how individuals manage the psychological and physical lines that separate their professional and personal lives. The theory proposes that well-being relies on the effective management of these boundaries, which are defined by their flexibility (the degree to which they can be adjusted) and permeability (the degree to which elements from one domain can enter another) (Ashforth et al., 2000; Clark, 2000; Kreiner, 2006). People develop segmentation or integration preferences: some prefer a clear separation between work and home, while others are comfortable with a more blended existence. However, these individual preferences are not always realized, as organizational culture and technology can dictate the actual nature of the boundary (Kreiner et al., 2009; Nippert-Eng, 1996).

The erosion of teacher WLB in the digital age is therefore best understood as a crisis of boundary management. Mobile technologies, particularly super-apps like WeChat, increase the permeability of the work-life border by allowing professional demands to flow into the home at all hours (Derks et al., 2014; Tian, 2020). While boundary theory acknowledges that individuals can employ “boundary work tactics” to protect their personal space, such as muting notifications or maintaining separate devices (Kreiner et al., 2009), these tactics are often neutralized by structural expectations of availability. In the Chinese educational context, the “digital panopticon” creates a condition where the boundary is not merely permeable but is forced into a state of permanent integration, overriding the teacher’s segmentation preferences and leading to “boundary thinning”—a chronic state where the line between work and life effectively disappears (Wu et al., 2025).

The theory thus helps to explain the mechanism behind the quantitative findings in this study. The mediating role of WLB in the relationship between PsyCap, burnout, and well-being is, in effect, a story about boundary management. High PsyCap may fail to protect well-being when the boundary is structurally non-negotiable, while burnout depletes the energy needed for any boundary maintenance. Furthermore, the “competence trap” identified in the qualitative phase can be reframed as a specific boundary failure: high self-efficacy leads teachers to voluntarily increase boundary permeability by accepting more work, making their personal domain more vulnerable. Ultimately, Boundary Theory offers a crucial lens for interpreting how “digital involution” fractures the separation between life domains, turning the smartphone from a tool of convenience into a mechanism of permanent observability that undermines the psychological detachment essential for recovery and well-being (Ashforth et al., 2000; Clark, 2000; Sonnentag and Fritz, 2015).

## 3 Method

This study employed an explanatory sequential mixed-methods design (Creswell and Plano Clark, 2017), comprising a primary quantitative phase followed by a secondary qualitative phase. In the first phase, cross-sectional survey data were collected and analyzed using Structural Equation Modeling (SEM) to test the hypothesized mediation model. In the second phase, we utilized the Critical Incident Technique (CIT; Flanagan, 1954) through targeted interviews. This approach was chosen to move beyond general self-reflections and examine the behavioral mechanisms through which PsyCap and burnout interact to influence work-life balance and well-being within specific, high-pressure professional contexts.

### 3.1 Participants and procedure

The target population comprised primary and secondary school teachers across three provinces in eastern and central China (Zhejiang, Jiangsu, and Henan), representing a mix of urban and rural educational settings. To ensure sample representativeness, a stratified random sampling technique was utilized based on school level (primary vs. secondary) and location (urban vs. rural).

Ethical approval was obtained from the university’s IRB prior to data collection. Surveys were distributed via school administrators using the secure Wenjuanxing platform. To enable the sequential mixed-methods design while safeguarding confidentiality, a two-stage consent and pseudonymization process was used. Participants first consented to a potential follow-up interview, then created a unique personal code (composed of their mother’s surname initials, birth year last two digits, and school initials) instead of providing their real name. This code linked survey responses to follow-up invitations, while contact details were stored separately in an encrypted file. Participants were assured confidentiality, aggregate reporting, and no right/wrong answers. This process allowed follow-up identification while strictly protecting privacy (Podsakoff et al., 2012).

Of the 1,050 surveys distributed, 894 were returned. After screening for incomplete responses, straight-lining, and multivariate outliers (assessed via Mahalanobis distance), a final valid sample of 842 teachers was retained (effective response rate = 80.2%). The sample was predominantly female (71.5%, *n* = 602), reflective of the teaching demographic in China. The mean age was 36.4 years (SD = 8.2), with an average teaching tenure of 12.1 years (SD = 7.5). Participants included primary school teachers (45.1%) and secondary school teachers (54.9%).

Following the quantitative analysis, a purposive sampling strategy was employed to recruit 20 teachers from the initial cohort who had consented to follow-up interviews and for whom a pseudonymous code was available. Participants were selected using a “deviant case” approach based on their standardized residuals from the structural model. The research team used the pseudonymous codes to identify the target cases and retrieve their contact information from the encrypted file. This strategy ensured that the qualitative data captured behavioral nuances in instances where the quantitative predictors (PsyCap and Burnout) failed to account for well-being outcomes. Specifically, we recruited two outlier profiles: (1) positive outliers, who maintained high work-life balance and well-being despite reporting high burnout or low PsyCap, and (2) negative outliers, who reported low well-being and significant boundary thinning despite possessing high PsyCap. To ensure transparent selection, we applied a two-stage filtering procedure. First, we excluded any of the 42 deviant cases with outdated contact information or who declined re-consent. Second, we ranked the remaining eligible cases within each outlier profile by the absolute magnitude of their standardized residuals, then selected the 11 teachers with the largest positive residuals (“resilient”) and the 9 with the largest negative residuals (“failed”). This maintained a proportional distribution close to the original breakdown (23 positive and 19 negative outliers) while prioritizing the most extreme cases. To preserve heterogeneity, we also ensured the final 20 included a mix of urban and rural schools and both primary and secondary teachers. This targeted selection allowed the study to identify the structural and digital factors that either neutralize personal resources or facilitate resilience in ways the SEM could not capture. The qualitative sample consisted of 14 females and 6 males, with a mean age of 38.2 years.

### 3.2 Measures

All self-report instruments were administered in Mandarin Chinese. To ensure consistency and contextual appropriateness for the teacher sample, we translated all scales anew using a rigorous back-translation procedure (Brislin, 1980) conducted by two bilingual researchers, with discrepancies resolved through consensus. No pre-existing Chinese versions were used. The construct validity of each translated scale was confirmed through separate Confirmatory Factor Analyses (CFAs); all measures demonstrated acceptable to excellent fit (e.g., CFI > 0.90, RMSEA < 0.08, SRMR < 0.08), as detailed below.

#### 3.2.1 Teacher burnout

Burnout was assessed using the Chinese translation of the Maslach Burnout Inventory-Educators Survey (MBI-ES; Maslach et al., 1996). This 22-item scale measures three dimensions: Emotional Exhaustion (9 items; e.g., “I feel emotionally drained from my work”), Depersonalization (5 items), and Personal Accomplishment (8 items, reverse-coded). Responses were recorded on a 7-point Likert scale ranging from 0 (*never*) to 6 (*every day*). Higher scores indicated higher levels of burnout. In the current study, the scale demonstrated excellent internal consistency (Cronbach’s *α* = 0.89). The CFA for the MBI-ES indicated a good fit to the data: χ^2^/df = 2.85; CFI = 0.951; TLI = 0.940; RMSEA = 0.052 [90% CI: 0.045, 0.059]; SRMR = 0.041.

#### 3.2.2 Psychological capital

Psychological capital (PsyCap) was assessed with the 24-item Psychological Capital Questionnaire (PCQ-24; Luthans et al., 2015). The instrument evaluates four dimensions: Self-Efficacy, Hope, Resilience, and Optimism, with six items each. Sample items include “I feel confident analyzing a long-term problem to find a solution” (Self-Efficacy) and “When I have a setback at work, I recover quickly” (Resilience). Ratings were assigned on a 6-point Likert scale ranging from 1 (*strongly disagree*) to 6 (*strongly agree*). Higher total and subscale scores indicated greater PsyCap. The PCQ-24 has proven validity across settings and cultures. In this sample, the overall scale showed high reliability (Cronbach’s *α* = 0.92), with subscales demonstrating robust consistency: Self-Efficacy (*α* = 0.88), Hope (*α* = 0.85), Resilience (*α* = 0.87), and Optimism (*α* = 0.84). The CFA for the PCQ-24 demonstrated a strong fit: χ^2^/df = 2.91; CFI = 0.962; TLI = 0.953; RMSEA = 0.048 [90% CI: 0.041, 0.055]; SRMR = 0.039.

#### 3.2.3 Work-life balance

The mediating variable, work-life balance, was evaluated using the 15-item Work-Life Balance Scale designed by Hayman (2005). This multifaceted scale consists of three distinct subscales: Work Interference with Personal Life (WIPL; 7 items), Personal Life Interference with Work (PLIW; 4 items), and Work-Personal Life Enhancement (WPLE; 4 items). Items were scored on a 7-point Likert scale (1 = *not at all*, 7 = *all the time*). Following standard scoring protocols, WIPL and PLIW items were reverse-coded so that higher overall scores reflected better work-life balance. The reliability for the overall scale in this study was strong (*α* = 0.86). The CFA for the WLB scale showed a good fit: χ^2^/df = 2.74; CFI = 0.948; TLI = 0.935; RMSEA = 0.055 [90% CI: 0.047, 0.063]; SRMR = 0.045.

#### 3.2.4 Psychological well-being

To gain a holistic understanding of the teachers’ mental well-being, we employed a shortened version of the Psychological Well-being Scale (PWBS) developed by Ryff and Keyes (1995). This 18-item instrument delves into six key facets of psychological well-being (Autonomy, Environmental Mastery, Personal Growth, Positive Relations, Purpose in Life, and Self-Acceptance), with each facet measured by three statements. Participants indicated their level of agreement on a 7-point scale, ranging from 1 (*strongly disagree*) to 7 (*strongly agree*). To ensure higher scores reflected greater well-being, negative items were reverse-coded during scoring. By examining these six facets, we gained a comprehensive understanding of the psychological well-being of the teacher population in this study. The scale yielded a Cronbach’s *α* of.88. The CFA for the PWBS indicated an acceptable fit: χ^2^/df = 3.12; CFI = 0.934; TLI = 0.921; RMSEA = 0.061 [90% CI: 0.054, 0.068]; SRMR = 0.049.

#### 3.2.5 Control variables

Based on previous literature linking demographic factors to occupational well-being, age, gender (0 = male, 1 = female), and teaching tenure (in years) were included as control variables in the structural models.

#### 3.2.6 Qualitative phase: critical incident protocol

Semi-structured interviews were replaced with a formal CIT protocol to capture concrete behavioral data. Interviews were conducted virtually via Tencent Meeting, lasting between 45 and 60 min. Instead of asking for general opinions on work-life balance, the protocol required each participant to reconstruct two “critical incidents” from the preceding 3 months: (1) a situation where they successfully navigated a severe conflict between professional duties and personal life, and (2) a situation where work-life conflict led to a perceived failure in maintaining well-being.

For each incident, the researcher utilized a retrospective probing technique to map the “anatomy of the event”: the specific trigger, the immediate cognitive and emotional response, the actions taken, and the ultimate outcome. Once the incident was reconstructed, specific probes were used to identify the activation (or absence) of PsyCap dimensions. For instance, to assess Resilience, teachers were asked: “At the moment the pressure peaked, what specific thought or resource allowed you to bounce back and maintain your boundary?” To assess Hope, we asked: “What specific pathways or alternative plans did you generate when your original schedule was disrupted by school demands?” This incident-based approach provided a microscopic view of how personal resources function as a buffer in real-time, reducing the risk of social desirability bias common in general well-being surveys.

### 3.3 Data analysis

Quantitative data were analyzed using SPSS 28.0 and Mplus 8.4. First, descriptive statistics, bivariate correlations, and reliability estimates were computed. Missing data (accounting for < 1% of data points) were handled using Full Information Maximum Likelihood (FIML). To statistically assess Common Method Bias, Harman’s single-factor test was conducted, and an unmeasured latent method factor was added to the measurement model; both tests confirmed that CMB did not significantly threaten the validity of the results.

The hypotheses were tested using a two-step Structural Equation Modeling (SEM) approach (Anderson and Gerbing, 1988). Initially, a measurement model using Confirmatory Factor Analysis (CFA) was specified to assess the construct validity and model fit of the latent variables. Fit indices included the Comparative Fit Index (CFI ≥ 0.90), Tucker-Lewis Index (TLI ≥ 0.90), Root Mean Square Error of Approximation (RMSEA ≤ 0.08), and Standardized Root Mean Square Residual (SRMR ≤ 0.08). Subsequently, the structural model was evaluated to test the direct and indirect paths. Mediation was tested using 5,000 bootstrap resamples to generate 95% bias-corrected confidence intervals; an indirect effect was considered significant if the confidence interval did not include zero.

Qualitative data derived from the CIT were analyzed using a hybrid thematic approach. First, each reported incident was treated as the primary unit of analysis and categorized based on the “effectiveness” of the outcome (Flanagan, 1954). We then applied Reflexive Thematic Analysis (Braun and Clarke, 2021) to code the transcripts, specifically looking for the “behavioral markers” of Hope, Efficacy, Resilience, and Optimism within the incidents. To ensure the rigor and trustworthiness of the coding process, two researchers independently coded the full set of transcripts. After completing their initial coding, the two coders met to compare their code assignments and discuss any discrepancies. Disagreements were resolved through consensus-based discussion, with reference back to the original transcripts and the operational definitions of the PsyCap dimensions. This iterative process led to a final set of agreed-upon codes, and the two coders then collaboratively grouped these codes into the overarching meta-themes reported in the qualitative results. Finally, a joint display matrix was created to integrate these incident-based behavioral markers with the quantitative paths. This allowed for a highly nuanced mixed-methods interpretation, illustrating not just *that* PsyCap mediates well-being, but *how* it is enacted during the specific work-life “friction points” unique to the Chinese educational system.

## 4 Results

### 4.1 Data screening and preliminary analyses

Prior to formal hypothesis testing, we conducted data cleaning and screening. Missing data (less than 1.2% of the total dataset) were found to be missing completely at random (MCAR) via Little’s MCAR test (*χ*^2^ = 42.15, *df* = 38, *p* = 0.296). Consequently, we utilized Full Information Maximum Likelihood (FIML) to handle missingness. Univariate normality was confirmed, with skewness values ranging from −0.84 to 1.12 and kurtosis from −0.72 to 1.45, both within the acceptable range of ±2 (Kline, 2023). Multivariate normality was further verified through Mardia’s coefficient (22.14, *p* < 0.05), suggesting that while there was slight multivariate non-normality, the use of maximum likelihood estimation with robust standard errors (MLR) in Mplus was appropriate.

To assess Common Method Bias (CMB), we first performed Harman’s single-factor test, where the first factor accounted for only 24.8% of the variance, well below the 50% threshold. Following Podsakoff et al. (2012), we then applied the unmeasured latent method factor (ULMF) approach. Comparing the measurement model with and without the method factor showed that the inclusion of the ULMF did not significantly improve fit (*Δ*CFI = 0.014, *Δ*TLI = 0.011, *Δ*RMSEA = 0.003), and the average variance explained by the method factor was 16.2%. These results suggest that CMB was unlikely to contaminate the findings.

Table 1 provides the means, standard deviations, and zero-order correlations. All variables exhibited significant correlations in the expected directions. Notably, we included the Heterotrait-Monotrait (HTMT) ratios to ensure discriminant validity; all HTMT values were below 0.85, confirming that the constructs are empirically distinct.

**Table 1:** Descriptive statistics, reliability, and construct validity.

Variable	M	SD	α	CR	AVE	1	2	3	4
1. Teacher burnout	3.42	1.15	0.89	0.91	0.64	—	0.48	0.41	0.51
2. PsyCap	4.12	0.82	0.92	0.93	0.68	−0.42***	—	0.52	0.61
3. Work-life balance	3.88	1.04	0.86	0.88	0.59	−0.38***	0.46***	—	0.55
4. Psych. well-being	4.55	0.92	0.88	0.90	0.62	−0.45***	0.54***	0.49***	—

Inter-construct correlations are below the diagonal; HTMT ratios are above the diagonal. ***p < 0.001.

### 4.2 Measurement model and alternative model testing

We used a two-step approach for Structural Equation Modeling (Anderson and Gerbing, 1988). Initially, we compared our hypothesized four-factor measurement model against three plausible alternative models to establish structural distinctiveness (see Table 2). The hypothesized four-factor model (Burnout, PsyCap, WLB, PWB) provided a significantly better fit to the data than the three-factor model (where WLB and PWB were merged) or the one-factor model.

**Table 2:** Comparison of measurement models.

Model	*χ* ^2^	df	*χ*^2^/df	CFI	TLI	RMSEA	SRMR
M1: Four-factor	312.45	104	3.00	0.958	0.949	0.049	0.038
M2: Three-factor^a^	845.22	107	7.90	0.882	0.861	0.092	0.074
M3: One-factor^b^	1654.89	110	15.04	0.721	0.684	0.134	0.112

a Burnout, PsyCap, and a merged WLB/PWB factor.

b All items loaded on a single latent factor.

### 4.3 Structural equation modeling (SEM)

After controlling for age, gender, and teaching tenure, the structural model demonstrated excellent fit: *χ*^2^(142) = 398.12, *p* < 0.001; CFI = 0.952; TLI = 0.941; RMSEA = 0.046, 90% CI [0.041, 0.052]; SRMR = 0.042.

**Figure 1:**
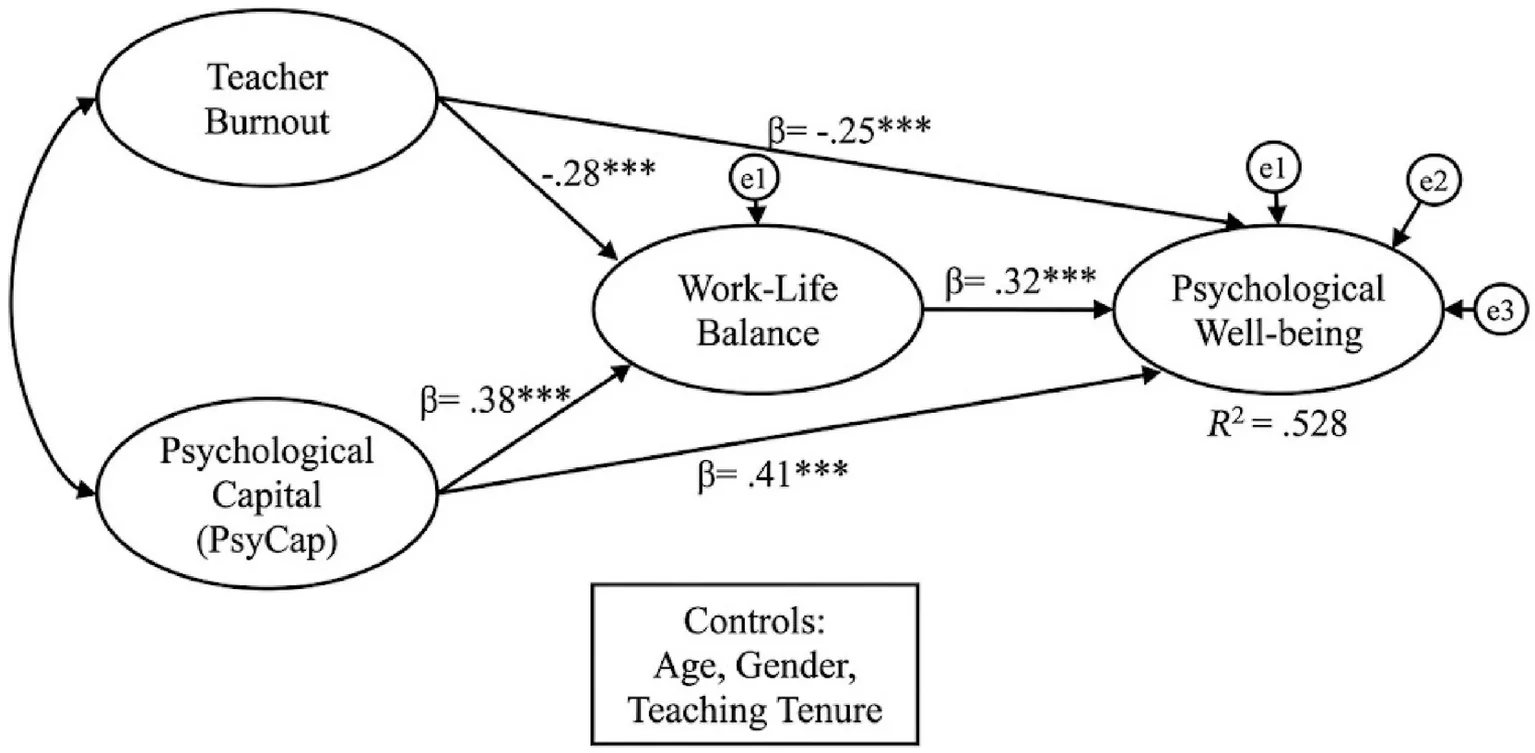
Structural equation model illustrating the mediating role of work-life balance. ***indicates statistical significance at the *p* < 0.001 level.

As seen in Figure 1, teacher Burnout was a robust negative predictor of WLB (*β* = −0.28, *SE* = 0.04, *p* < 0.001) and Well-being (*β* = −0.25, *SE* = 0.05, *p* < 0.001). PsyCap was the strongest positive predictor of Well-being (*β* = 0.41, *SE* = 0.04, *p* < 0.001) and was positively associated with WLB (*β* = 0.38, *SE* = 0.03, *p* < 0.001). WLB, in turn, predicted Well-being (*β* = 0.32, *SE* = 0.04, *p* < 0.001). The control variables exerted minimal influence, though tenure showed a weak positive association with burnout (*β* = 0.14, *p* = 0.008). The model explained 52.8% of the variance in Psychological Well-being.

### 4.4 Mediation and residual analysis for qualitative selection

To test the indirect effects, we used a bias-corrected bootstrapping procedure (5,000 samples). Table 3 presents a full breakdown of the mediation analysis, disaggregating the total effect for each relevant predictor into its direct and indirect components. The table explicitly lists the direct path coefficients for the connections between the predictors and the mediator, as well as between the mediator and the outcome. As shown in the table, Work-Life Balance significantly mediated the associations between both predictors and Psychological Well-being, with neither bootstrapped confidence interval containing zero.

**Table 3:** Direct, indirect, and total effects for the structural pathways.

Structural path	Direct effect (*β*)	Indirect effect (*β*)	Total effect (*β*)	95% CI (Indirect)
Direct effects				
Burnout → WLB	−0.28***	—	−0.28***	—
PsyCap → WLB	0.38***	—	0.38***	—
WLB → PWB	0.32***	—	0.32***	—
Burnout → PWB	−0.25***	—	−0.25***	—
PsyCap → PWB	0.41***	—	0.41***	—
Mediated effects				
Burnout → WLB → PWB	—	−0.090***	−0.340***	[−0.128, −0.057]
PsyCap → WLB → PWB	—	0.122***	0.532***	[0.079, 0.174]

Bootstrap resamples = 5,000; PWB, psychological well-being; ***p < 0.001. All models controlled for age, gender, and teaching tenure.

To prepare for the qualitative phase, we conducted a residual analysis of the structural model to identify cases where the observed scores for well-being and balance differed significantly from the values predicted by the model. By calculating the standardized residuals for the path between PsyCap and Well-being, we identified departures from the hypothesized resource-pathway logic. While the model showed strong predictive power, we identified 42 “deviant cases” (standardized residuals > ± 2.0). Applying the two-stage selection criteria detailed in the Method section (which considered contact availability, confirmed consent, and the magnitude of the residuals), we finalized a qualitative sample of 20 participants. These included 11 “resilient” teachers who maintained stability despite high burnout and 9 teachers who experienced well-being failure despite high personal resources. This approach provided a microscopic view of how specific friction points—such as the “competence trap”—prevent personal resources from functioning as effective buffers when analyzed via the Critical Incident Technique.

### 4.5 Qualitative results: critical incident analysis

The qualitative phase sought to unpack the “partial mediation” observed in the structural model by examining 40 specific critical incidents (20 “successful” resolutions and 20 “failed” resolutions) reported by participants. Analysis revealed three overarching meta-themes that explain the behavioral enactment of PsyCap and the unique cultural barriers to Work-Life Balance (WLB) in the Chinese educational context.

#### 4.5.1 Tactical pathway generation vs. passive optimism

While the quantitative results identified Hope as a significant predictor, the CIT analysis revealed a clear distinction between Passive Optimism (believing things will improve) and Tactical Pathway Generation (the ability to create alternative routes to goals).

In “successful” incidents, teachers high in the *Hope* dimension did not just “stay positive”; they proactively renegotiated boundaries. Participant 07 (High PsyCap/High WLB) illustrated this during a conflict where a mandatory weekend competition overlapped with a long-planned family reunion. Rather than resigning herself to the workload, she explained that she “did not just hope the school would understand,” but instead “prepared a detailed digital resource pack” for her students to use independently. By negotiating a shift swap with a colleague, she noted that the goal was not necessarily to feel “happy,” but rather to find a “strategic loop-hole” that successfully protected her personal time.

In contrast, teachers with lower *Hope* scores often viewed workplace demands as immutable. Participant 12 (Low PsyCap/Low WLB) described a similar weekend assignment as an “unavoidable fate,” stating that “questioning the schedule feels like questioning the system itself.” This lack of pathway thinking led to a passive acceptance of work-life conflict, where the teacher felt there was “simply no other way to exist in this school” (P12). These narratives suggest that high burnout effectively paralyzes the capacity for pathway generation, which explains why the quantitative path from Burnout to WLB remained so robust.

#### 4.5.2 The “digital panopticon” and boundary thinning

The qualitative data highlighted a specific mechanism of Work Interference with Personal Life (WIPL) that the survey could not capture: the role of instant messaging (WeChat) as a tool of “Digital Involution” (*Neijuan*).

Seventeen out of 20 teachers described critical incidents where their WLB was fractured by the expectation of 24/7 digital availability. Participant 14 (Low PsyCap/High Burnout) recalled a parent’s message at 10:30 p.m. regarding a minor homework error, describing the notification sound as “an electric shock.” She noted that even if she chose not to reply, the sense of “personal growth” usually felt at home was “replaced by a sense of being watched.” She emphasized that it is impossible to “switch off resilience” when the boundary is an “invisible, vibrating phone in your pocket” (P14).

Other participants echoed this sense of digital surveillance. Participant 18 (High Burnout) described the pressure to respond to school group chats immediately as a “silent rule” that made his home feel like “an extension of the staff room.” He remarked that “silencing the phone feels like a dereliction of duty,” illustrating how the facet of Environmental Mastery—a key part of well-being—is eroded by digital encroachment. This confirms that resilience in this context is often “reactive”; teachers are using their psychological energy simply to endure constant boundary thinning rather than proactively preventing it.

#### 4.5.3 The paradox of high efficacy: the “competence trap”

An unexpected finding that sheds light on the negative SEM residuals was the “Competence Trap.” This theme represents a paradoxical mechanism wherein high levels of self-efficacy—usually a protective asset—fail to sustain well-being under conditions of extreme institutional demand. Teachers who scored high on Self-Efficacy in the quantitative phase often reported the most severe burnout during interviews.

The data suggest that believing “I can handle any task” leads high-efficacy teachers to accept excessive “invisible labor.” Participant 03 (High PsyCap/High Burnout) reflected on a failed incident where she took on three additional graduating classes, driven by the belief that she was “the only one who could get these students through the Gaokao.” However, this confidence became a “trap,” as she admitted that while she was “confident in managing the work,” she ended up “ignoring the total collapse” of her own life. This resulted in a hollow sense of achievement; although she succeeded professionally, she noted that her “purpose in life felt hollow” because she had not seen her own children for a week (P03). This narrative illustrates how high efficacy can function as a boundary condition within involuted systems: when institutional demands are perceived as unlimited, individual confidence may lead to a dangerous over-extension of personal resources (Bandura, 1997; Karasek and Theorell, 1990).

This “trap” extended to emotional labor as well. Participant 05 (High Efficacy) described a situation where she spent hours after school counseling students, not because it was required, but because she felt she was “uniquely capable of solving their problems.” She reflected that “being good at your job makes it harder to say no to more work,” eventually leading to a state where “efficacy fuels exhaustion” (P05). These qualitative insights provide a nuanced explanation for the negative residuals observed in the structural model. While the broader quantitative trend remains positive and linear, the “Competence Trap” identifies a specific friction point where extreme efficacy in a high-pressure system can paradoxically accelerate burnout by encouraging teachers to accept labor that exceeds their recovery capacity.

### 4.6 Joint display matrix: integration of quantitative and qualitative findings

Following best practices for mixed-methods reporting (Creswell and Plano Clark, 2017), we constructed a joint display matrix to visually demonstrate how the qualitative findings elaborate on and explain the quantitative pathways identified in the SEM. Table 4 presents the integration of the three meta-themes derived from the Critical Incident Technique with the corresponding quantitative paths from the structural model.

**Table 4:** Joint display matrix: Integration of quantitative paths and qualitative themes.

Quantitative path	Qualitative theme	Illustrative CIT evidence	Integration and interpretation
PsyCap → WLB (*β* = 0.38, *p* < 0.001)	Tactical Pathway Generation vs. Passive Optimism	P07: “prepared a detailed digital resource pack” and negotiated a “shift swap” to protect personal time. P12: viewed weekend assignment as “unavoidable fate,” lacking pathway thinking.	High PsyCap is effective when enacted as proactive problem-solving (pathway generation), not passive optimism. When teachers generate alternative routes to manage work demands, they successfully protect WLB. Low PsyCap manifests as pathway paralysis, where teachers accept work-life conflict as immutable.
Burnout → WLB (*β* = −0.28, *p* < 0.001)	Tactical Pathway Generation vs. Passive Optimism	P12: “questioning the schedule feels like questioning the system itself.” P07: actively sought “strategic loop-hole” despite high demands.	Burnout depletes the cognitive and motivational resources needed for pathway generation. Exhausted teachers perceive the system as non-negotiable, which explains the robust negative path from burnout to WLB. The qualitative data show that burnout operates by paralyzing agency rather than merely increasing workload.
PsyCap → PWB (*β* = 0.41, *p* < 0.001)	The “Digital Panopticon” and Boundary Thinning	P14: notification sound as “an electric shock”; “impossible to switch off resilience.” P18: home feels like “extension of the staff room.”	PsyCap’s protective effect on well-being is constrained by digital permeability. Even high-PsyCap teachers struggle when boundaries are structurally forced into permanent integration. The qualitative data show that “reactive resilience” (enduring constant boundary thinning) differs from “proactive resilience” (preventing boundary violation).
Burnout → PWB (*β* = −0.25, *p* < 0.001)	The “Digital Panopticon” and Boundary Thinning	P18: “silencing the phone feels like a dereliction of duty.” P14: “personal growth replaced by sense of being watched.”	Burnout is exacerbated by digital surveillance because teachers cannot psychologically detach from work. The anticipatory stress of potential notifications prevents recovery, directly impairing the Environmental Mastery facet of well-being.
WLB → PWB (*β* = 0.32, *p* < 0.001)	All three themes converge on WLB as the mechanism	Successful cases (P07, P11) maintained WLB through tactical pathway generation despite high demands. Failed cases (P03, P05) experienced boundary collapse despite high PsyCap.	WLB functions as the proximal mechanism through which both PsyCap and burnout influence well-being. When WLB is protected through pathway generation, well-being is sustained even under high demands. When WLB is eroded by digital permeability or the competence trap, well-being declines regardless of personal resources.
PsyCap → PWB (Residual analysis: negative outliers)	The “Competence Trap”	P03: “confident in managing the work” but “ignoring the total collapse” of personal life. P05: “being good at your job makes it harder to say no to more work.”	The competence trap explains the negative SEM residuals where high-PsyCap teachers reported unexpectedly low well-being. High self-efficacy paradoxically accelerates burnout by encouraging acceptance of excessive invisible labor. Confidence becomes a liability when institutional demands are unlimited and boundary-setting is culturally discouraged.
Burnout → PWB (Residual analysis: positive outliers)	Tactical Pathway Generation (protective factor)	“Resilient” teachers (P07, P11) maintained well-being despite high burnout by generating strategic alternatives to protect personal time.	Positive residuals—where teachers maintained well-being despite high burnout—were explained by tactical pathway generation. These teachers used “strategic loop-holes” to preserve WLB, suggesting that pathway thinking can partially offset the health-impairment process of burnout.

Overall, the matrix reveals three integrative patterns. First, the mediating role of WLB is explained by tactical pathway generation: PsyCap predicts WLB through proactive problem-solving, while burnout reflects pathway paralysis. Second, the digital panopticon explains why WLB only partially mediates the paths to well-being—digital permeability creates boundary thinning that operates independently of perceived balance. Third, the competence trap provides a behavioral explanation for negative residuals among high-PsyCap teachers: self-efficacy paradoxically accelerates burnout by encouraging over-extension. Together, these findings demonstrate that the qualitative phase not only confirms but also enriches the quantitative results, offering a more complete understanding of teacher well-being in the Chinese educational context.

## 5 Discussion

The present study employed an explanatory sequential mixed-methods design to investigate the relationships between teacher burnout, psychological capital (PsyCap), and psychological well-being (PWB) among Chinese educators, with a focus on the mediating role of work-life balance (WLB). The quantitative findings provide robust evidence that WLB serves as a critical bridge through which personal resources and occupational stressors influence overall well-being. This dynamic was reflected in the structural analyses, which demonstrated that WLB mediates the effects of both burnout and PsyCap on teacher well-being. This pattern aligns with the Job Demands-Resources (JD-R) model, supporting the idea that working conditions initiate a dual process of health-impairment and motivation (Bakker and Demerouti, 2017; Schaufeli and Taris, 2014). Within this framework, our findings suggest burnout functioned as a potent drain on the energetic reserves required for boundary maintenance (Maslach and Leiter, 2016). Specifically, the negative link from burnout to WLB suggests that emotional exhaustion and depersonalization do not remain trapped as internal states but instead spill over into the personal domain, eroding the teacher’s capacity for recovery (Sonnentag and Fritz, 2015). On the other hand, PsyCap served as a meaningful predictor of both WLB and PWB, bolstering Conservation of Resources (COR) theory by suggesting that teachers with high PsyCap possess a “resource caravan” (Hobfoll et al., 2018) that allows them to negotiate life roles more effectively. However, the presence of direct effects alongside the mediated pathways indicates that other factors—perhaps direct physiological effects of stress or organizational climate—also contribute to the well-being of Chinese educators (Angelini et al., 2024; Md Shah et al., 2024). It is also important to interpret the relative magnitude of these effects. Although the indirect pathways through WLB were statistically significant, their standardized coefficients were modest (burnout *β* = −0.090; PsyCap *β* = 0.122). In contrast, the direct effects were substantially larger (burnout *β* = −0.25; PsyCap *β* = 0.41). This discrepancy implies that WLB serves as a meaningful but secondary bridge, rather than the dominant explanatory mechanism. Practically, this means that teacher well-being is primarily shaped by the direct psychological toll of occupational stress and the direct protective benefits of PsyCap, rather than being funneled exclusively through the preservation of work-life boundaries. Consequently, interventions designed solely to improve balance may produce limited gains unless they also address the core drivers of exhaustion and directly strengthen personal psychological resources.

To move beyond these statistical trends, the qualitative Critical Incident Technique (CIT) analysis was used to uncover the structural and digital mechanisms—namely the “Digital Panopticon” and the “Competence Trap”—that govern how these resources are enacted or depleted in the era of involution. The identification of this “Digital Panopticon” is a particularly significant driver of boundary thinning. While traditional Boundary Theory (Ashforth et al., 2000; Kreiner, 2006) emphasizes individual integration or segmentation preferences, the CIT data suggests that in this specific context, personal preferences are often overridden by “super-apps” like WeChat (Tian, 2020; Yu, 2024). Participants described a state of permanent observability where the smartphone acts as a “24-h digital sentry post,” leading to a state of “recovery obstruction” (Derks et al., 2014). In such a system, the anticipatory stress of parent or administrator messages prevents true psychological detachment, and even teachers with high psychological resources struggle with the presence of the “invisible, vibrating phone.” This indicates that personal resilience is often neutralized by structural expectations of 24/7 availability (Wu et al., 2025), forming a localized form of “Digital Involution” where the pressure to remain digitally responsive becomes a defensive competition that yields diminishing returns on well-being (Xia et al., 2025).

Another striking finding revolved around the “Competence Trap” identified in the qualitative phase. While PsyCap theory generally views self-efficacy as a protective buffer (Luthans et al., 2015), the current data reveals a U-shaped or paradoxical relationship in high-pressure environments. Notably, highly efficacious teachers reported taking on excessive “invisible labor”—such as intensive Gaokao counseling or administrative tasks—driven by the belief that they were uniquely capable (Bandura, 1997; Karasek and Theorell, 1990). This “trap” suggests that high efficacy can lead to a dangerous over-extension of personal resources, where teachers prioritize professional “environmental mastery” at the expense of their own “purpose in life” and family relations (Ryff, 2014). Consequently, this provides a plausible explanation for the counterintuitive patterns in the data, where some highly resourceful teachers reported unexpectedly low well-being. In the context of involution, high competence often results in a “reward” of more work, creating a cycle where efficacy fuels exhaustion rather than mitigating it (Ye et al., 2026).

Although the terms “competence trap” and “digital panopticon” have been discussed separately (Karasek and Theorell, 1990; Tian, 2020; Ye et al., 2026; Yu, 2024), the present study extends these concepts in three key ways. First, we integrate them within a unified framework: the quantitative model identifies WLB as the critical pathway, while the CIT shows that the competence trap operates through boundary permeability—high-efficacy teachers accept more work, eroding WLB—and that this erosion is amplified by the digital panopticon’s constant demands. Second, our mixed-methods design provides real-time behavioral evidence from deviant cases, moving beyond theoretical speculation or cross-sectional correlations. Third, we contextualize both phenomena within Chinese *neijuan*, where competence invites more labor and digital surveillance is structurally enforced. Together, this integration offers a more complete understanding than prior studies that treated these concepts in isolation.

These structural pressures further explain why the “Hope” dimension of PsyCap is most effective when enacted as “Tactical Pathway Generation.” Unlike passive optimism, successful teachers in this study utilized “strategic loop-holes”—such as digital resource packs or shift swaps—to protect their WLB, a strategy that aligns with Hope Theory (Snyder, 2002). This demonstrates that “waypower” (pathway thinking) is just as critical as “willpower” (agency) for maintaining stability. Nevertheless, the qualitative results also identified “pathway paralysis” among resource-depleted teachers, who viewed the school system as a non-negotiable monolith (Merolla, 2014). The overall evidence suggests that for PsyCap to be effective, teachers must perceive their environment as having a degree of “situational negotiability” (Karasek and Theorell, 1990). Without institutional support or clear boundary rules, even the most hopeful teachers eventually succumb to the structural pressures of the Chinese educational landscape (Wang et al., 2025; Zhang and Li, 2025).

## 6 Conclusion and implications

This study concludes that the well-being of Chinese educators is no longer merely a product of individual resilience or classroom workload, but is increasingly dictated by the digital and structural boundaries—or lack thereof—in an era of intense educational involution. While psychological capital remains a vital internal asset, its role as a buffer is significantly mediated by the teacher’s ability to maintain a functional work-life balance. The transition of the school environment into a digital panopticon has fundamentally altered the nature of teacher labor, transforming the smartphone into a tool of permanent observability that prevents cognitive recovery. Most significantly, this research highlights that high professional efficacy can be a double-edged sword; in a system that rewards capability with more invisible labor, the most competent teachers are often the most at risk for total resource depletion. Ultimately, fostering teacher well-being in the modern age requires a move away from the expectation of unconditional self-sacrifice toward a model of sustainable professional engagement supported by clear digital boundaries.

The findings extend the Job Demands-Resources model by demonstrating that digital encroachment through super-apps creates a unique category of “boundary demands” that function differently from traditional physical workload. This study contributes to boundary theory by illustrating how cultural and technological pressures can render individual segmentation preferences irrelevant, creating a state of forced integration. Furthermore, the identification of the “competence trap” challenges the universal positivity of psychological capital, suggesting that in highly competitive environments, self-efficacy can paradoxically accelerate burnout by encouraging over-extension. Beyond these individual contributions, the study’s primary novelty is its integrative perspective: we show that the digital panopticon and the competence trap are not independent phenomena but interconnected forces that jointly erode well-being through the mediating mechanism of WLB. By using a mixed-methods design with deviant case sampling, we provide concrete behavioral evidence of how these traps operate in real-time, rather than merely describing them theoretically. This integration—coupled with the specific Chinese *neijuan* context—offers a more nuanced and actionable understanding than prior studies that examined each concept separately. This suggests a need for more context-sensitive theories of resilience that account for how personal strengths interact with systemic involution and digital surveillance.

For school leadership, the most pressing implication is the need to move beyond individualized resilience training and instead address the structural roots of exhaustion. Administrators should implement “right to disconnect” policies that establish clear offline hours for school WeChat groups, effectively dismantling the digital panopticon and allowing teachers true psychological detachment. Leadership training must also focus on the equitable distribution of “invisible labor” to ensure that high-performing teachers are not penalized with excessive responsibilities that lead to a competence trap. On an individual level, professional development should prioritize “tactical pathway generation” over passive optimism, equipping teachers with specific negotiation and digital management skills to proactively protect their personal time. Finally, policy interventions at the provincial level should reconsider evaluation metrics that fuel irrational competition, shifting the focus toward long-term educator health as a prerequisite for student success.

## 7 Limitations and suggestions for future research

Despite the insights gained from the mixed-methods approach, certain limitations provide a roadmap for future inquiry. First, while the qualitative phase utilized the Critical Incident Technique to capture concrete behaviors, the quantitative data remained cross-sectional, which limits the ability to draw firm causal conclusions about the long-term progression of the competence trap. Specifically, our identification of the competence trap relies on the assumption that high self-efficacy leads to over-extension and subsequent burnout. However, the cross-sectional design cannot rule out reverse causality—for example, that teachers already experiencing burnout may inflate their efficacy beliefs as a coping mechanism, or that the observed association reflects unmeasured third variables such as organizational culture. Additionally, the retrospective nature of the CIT data means that participants’ recollections of their efficacy and workload may be influenced by their current burnout levels, further complicating causal interpretation. Future researchers should employ longitudinal designs or diary studies to track how the relationship between efficacy and burnout evolves over the course of a high-stakes academic year. Second, this study relied on self-reported perceptions of digital interference; future work could benefit from objective digital metrics, such as logs of after-hours communication frequency, to provide a more precise measure of digital encroachment.

Additionally, the sample focused primarily on primary and secondary teachers in three provinces; expanding this research to higher education or vocational settings might reveal different boundary-management dynamics. It would also be valuable to investigate the role of gender and career stage more deeply, specifically examining whether novice teachers experience the digital panopticon differently than their veteran counterparts who may have more established professional authority. Finally, future research should explore the “recipient” side of the digital panopticon by interviewing parents and administrators to understand the systemic drivers of the expectation for 24/7 availability.

## Data Availability

The raw data supporting the conclusions of this article will be made available by the authors, without undue reservation.
